# Analysis of Bacterial Community Structure of Activated Sludge from Wastewater Treatment Plants in Winter

**DOI:** 10.1155/2018/8278970

**Published:** 2018-03-07

**Authors:** Shuang Xu, Junqin Yao, Meihaguli Ainiwaer, Ying Hong, Yanjiang Zhang

**Affiliations:** College of Resources and Environmental Science, Xinjiang University, Urumqi, China

## Abstract

Activated sludge bulking is easily caused in winter, resulting in adverse effects on effluent treatment and management of wastewater treatment plants. In this study, activated sludge samples were collected from different wastewater treatment plants in the northern Xinjiang Uygur Autonomous Region of China in winter. The bacterial community compositions and diversities of activated sludge were analyzed to identify the bacteria that cause bulking of activated sludge. The sequencing generated 30087–55170 effective reads representing 36 phyla, 293 families, and 579 genera in all samples. The dominant phyla present in all activated sludge were Proteobacteria (26.7–48.9%), Bacteroidetes (19.3–37.3%), Chloroflexi (2.9–17.1%), and Acidobacteria (1.5–13.8%). Fifty-five genera including* unclassified_f_Comamonadaceae*,* norank_f_Saprospiraceae*,* Flavobacterium*,* norank_f_Hydrogenophilaceae*,* Dokdonella*,* Terrimonas*,* norank_f_Anaerolineaceae*,* Tetrasphaera*,* Simplicispira*,* norank_c_Ardenticatenia*, and* Nitrospira* existed in all samples, accounting for 60.6–82.7% of total effective sequences in each sample. The relative abundances of Saprospiraceae,* Flavobacterium*, and* Tetrasphaera* with the respective averages of 12.0%, 8.3%, and 5.2% in bulking sludge samples were higher than those in normal samples. Filamentous Saprospiraceae,* Flavobacterium*, and* Tetrasphaera* multiplied were the main cause for the sludge bulking. Redundancy analysis (RDA) indicated that influent BOD_5_, DO, water temperature, and influent ammonia had a distinct effect on bacterial community structures.

## 1. Introduction

Activated sludge process has been extensively used in industrial and domestic wastewater treatment because of its high microbial diversity and activity, resulting in the removal of most organic pollutants and nutrients [1]. It is reported that the composition and diversity of the microbial community had the greatest impact on stability and performance of the wastewater treatment systems [2]. The biological community of activated sludge has a large biological diversity and contains a variety of viruses, bacteria, protozoa, fungi, algae, and metazoan. In this complex ecosystem, bacteria typically account for 95% of the total number of microbes and play a crucial part in wastewater treatment [3]. In the secondary clarifying pond, good compaction (thickening) and separation (settling) of the activated sludge has a positive effect on the effluent quality. However, bulking sludge due to overgrowth of filamentous bacteria and/or* Zoogloea* organisms has a significant influence on the performance of the activated sludge system as it can result in poor settling and poor compaction [4]. The overgrowth of filamentous bacteria of activated sludge has significantly affected the operation of wastewater treatment plants for many years [5]. Sludge bulking can easily occur at low temperature, resulting in adverse effects on effluent treatment [6]. Therefore, sludge bulking in winter caused by low temperature is the focus of the study.

The high-throughput sequencing technologies originated several years ago are easier and less expensive for high-throughput sequencing [7]. This method has been broadly used in the evaluation of microbial communities of many environmental samples types such as activated sludge [8, 9], marine water [10], soil [11], and human distal intestine [12]. Some studies have focused on the difference in microbial community composition of activated sludge due to temporal and spatial changes [13], wastewater characteristics [14], and environmental and operational conditions [15] in municipal wastewater systems. The microbial communities in activated sludge samples collected from wastewater treatment plants have been studied in different geographical location [16–18]. However, only few studies investigated the bacterial community compositions and diversities in various wastewater treatment plants (WWTPs) at different geographic locations in Xinjiang Uygur Autonomous Region of China via Illumina high-throughput sequencing technology, especially in winter.

The purpose of this study was to analyze bacterial community structures and diversities of activated sludge samples of different wastewater treatment plants in the north of Xinjiang Uygur Autonomous Region of China in winter via Illumina high-throughput sequencing technology. This study facilitates the evaluation of the similarities and differences in bacterial community composition of samples from different geographic locations and understanding the microbial interaction of activated sludge in Xinjiang.

## 2. Materials and Methods

### 2.1. Description of WWTPs and Sample Collection

Activated sludge samples were collected from the aeration tanks of four WWTPs located in the north of Xinjiang Uygur Autonomous Region of China in winter. These four WWTPs applied oxidation ditch process. The following 8 samples were collected: ALT1 and ALT2 from ALT WWTP; SHZ1 and SHZ2 from SHZ WWTP; CJ1 and CJ2 from CJ WWTP; HX1 and HX2 from HX WWTP. Sampling date, flow rate, influents, effluents, and operational parameters of the WWTPs are presented in Table 1. All WWTPs treat domestic wastewater, except for the SHZ WWTP. The influent of SHZ WWTP is composed of domestic and industrial wastewater, of which industrial wastewater accounts for 24%. SVI greater than 150 mL·g^−1^ is considered as bulking sludge [19]. Since the SVI of the samples from CJ and HX WWTPs were greater than 150 mL·g^−1^, the samples were bulking sludge, while the samples from ALT and SHZ WWTPs were normal sludge since their SVI were less than 150 mL·g^−1^ (Table 1). Additionally, the microscopic investigation showed that the sludge samples were filamentous bulking sludge.

The samples for the microbial analysis were stored in the laboratory at −40°C and were sent to Majorbio Bio-Pharm Technology Co. Ltd. (Shanghai, China) for DNA extraction, PCR amplification, and Illumina high-throughput sequencing.

### 2.2. DNA Extraction, PCR Amplification, and Illumina Sequencing

Microbial DNA was extracted from sludge samples collected from four WWTPs using the E.Z.N.A.® soil DNA Kit (Omega Bio-tek, Norcross, GA, US). The final DNA concentration and purification were determined by NanoDrop 2000 UV-vis spectrophotometer (Thermo Scientific, Wilmington, USA), whereas DNA quality was checked by 1% agarose gel electrophoresis. The V4-V5 hypervariable regions of the bacteria 16S r RNA gene were amplified with primers 515 F (5′-GTGCCAGCMGCCGCGG-3′) and 907 R (5′-CCGTCAATTCMTTTRAGTTT-3′) by thermocycler PCR system (GeneAmp 9700, ABI, USA). The PCR reactions were conducted using the following program: 3 min of denaturation at 95°C, 27 cycles of 30 s at 95°C, 30 s for annealing at 55°C, 45 s for elongation at 72°C, and a final extension at 72°C for 10 min. PCR reactions were performed in triplicate of 20 *μ*L mixture containing 4 *μ*L of 5x FastPfu Buffer, 2 *μ*L of 2.5 mM dNTPs, 0.8 *μ*L of each primer (5 *μ*M), 0.4 *μ*L of FastPfu Polymerase, and 10 ng of template DNA. The PCR products were extracted from a 2% agarose gel and further purified using the AxyPrep DNA Gel Extraction Kit (Axygen Biosciences, Union City, CA, USA). Then, the products were quantified using QuantiFluor™-ST (Promega, USA).

Purified amplicons were pooled in equimolar and paired-end sequenced (2  ×  300) on an Illumina MiSeq platform (Illumina, San Diego, USA) according to the standard protocols of Majorbio Bio-Pharm Technology Co. Ltd. (Shanghai, China). The raw reads were deposited into the NCBI Sequence Read Archive (SRA) database (Accession Numbers: SRP113278, SRP125654, and SRP126028).

### 2.3. Data Analysis

Data analysis was conducted using the i-Sanger platform (http://www.i-sanger.com/) provided by Majorbio Bio-Pharm Technology Co. Ltd. (Shanghai, China). The microbial phylotype richness levels were calculated using the Chao/Ace estimator and the Shannon diversity index. The similarity and difference of samples were compared using the shared and unique OTUs of Venn diagram. The species diversity of the ecosystem was compared using the rarefaction curves, which are the commonly used methods. Redundancy analysis (RDA) was used to analyze the relationship between the relative abundance of bacteria (genus level) and environmental variables, which is a type of constrained ordination. The Chao/Ace estimator, the Shannon diversity index, and the coverage percentage were also calculated by the Mothur program version v.1.30.1 (https://www.mothur.org/wiki/Schloss_SOP#Alpha_diversity). The similarity and differences between samples were compared using the shared and unique OTUs of the Venn diagram. The species diversity of the ecosystem was compared using the rarefaction curves, which are the commonly used methods. Redundancy analysis (RDA) was used to analyze the relationship between the relative abundance of bacteria (genus level) and environmental variables, which is a type of constrained ordination. These analyses were performed using the R Programming Language software.

## 3. Results and Discussion

### 3.1. Diversity Analysis for Bacterial Communities

As shown in Table 2, total effective reads of all activated sludge samples were 30087–55170. The microbial diversity index is listed in Table 2, comprising community richness (Ace, Chao) and community diversity (Shannon). Each sample was more than 99% of the coverage, indicating that the depth of the sequence was sufficient. According to the OTU number, the sample from SHZ WWTP had the richest diversity, followed closely by those from CJ and HX WWTP, whereas the sample from ALT WWTP displayed considerably less richness. According to Table 2, the values of Ace, Chao, and Shannon indices demonstrate that SHZ WWTP had the highest microbial diversity, while ALT WWTP had the lowest one.

**Figure 1:**
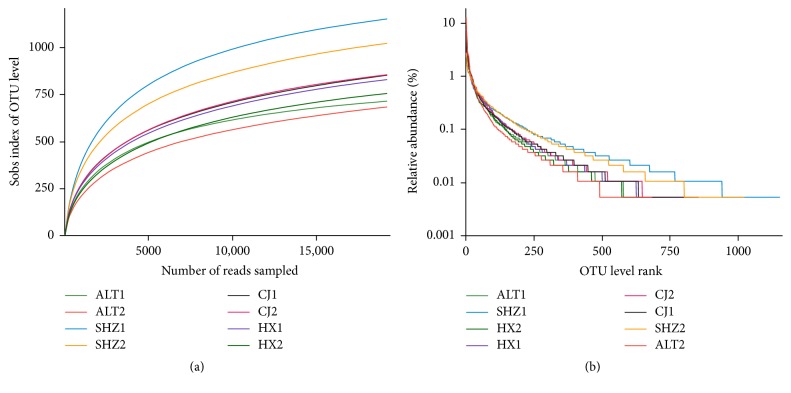
Diversity of bacterial communities in activated sludge samples. (a) Rarefaction curves and (b) rank abundance curves.

As shown in Figure 1(a), the rarefaction curves of these samples are approaching plateaus, indicating that highly diverse microbial communities were present in each sample. The distribution rarefaction curves and rank abundance curves also illustrated a much lower microbial diversity in ALT WWTP (Figure 1).

The ALT WWTP had the lowest microbial diversity, which can be because the temperature was lower than other WWTPs. Temperature has a decisive role in the metabolism of microorganisms, and lowering temperature has a significant effect on the reduction of the maximum specific growth [20]. In addition, wastewater types, industrial, domestic, and/or mixed, and their influent qualities have an important impact on microbial communities of activated sludge system [21]. The SHZ WWTP had the highest microbial diversity, which can be because the wastewater type was mixed.

### 3.2. Bacterial Community Composition and Similarity Analysis

**Figure 2:**
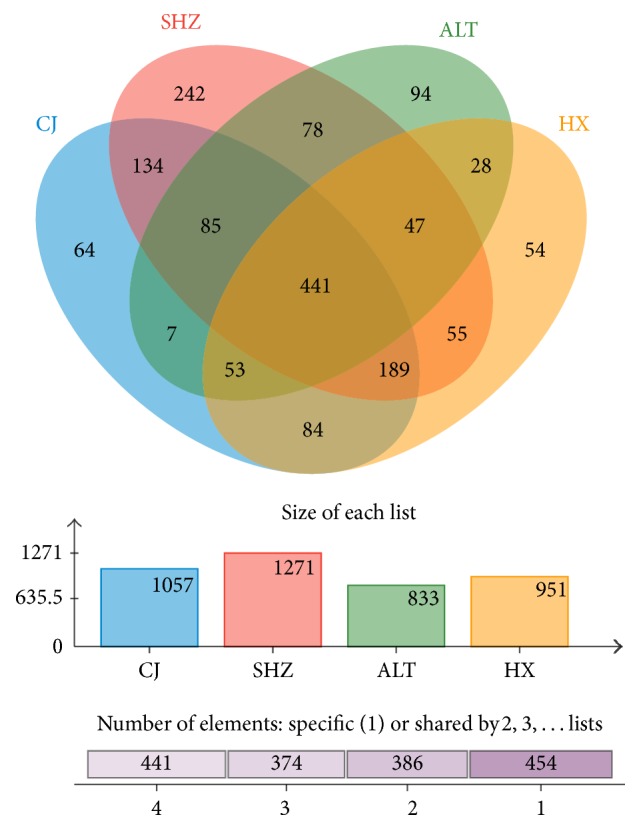
Overlap of the bacterial communities from four WWTPs based on OTU (3% distance).

The difference and similarity of bacterial community of activated sludge samples collected from different WWTPs were analyzed based on OTUs through the Venn diagram (Figure 2). The number of shared OTUs was 441 accounting for 26.6% of the total observed OTUs (1655). The shared OTUs indicated that some microorganisms always existed in the activated sludge collected from different WWTPs. In addition, the unique OTU numbers in sludge samples from ALT WWTP, SHZ WWTP, CJ WWTP, and HX WWTP were 94, 242, 64, and 54, respectively. The unique OTU number accounted for 3.3–5.7% with an average of 4.3%; the small quantity of unique microorganism appeared in the activated sludge, except for the SHZ WWTP (14.6%). The SHZ WWTP had a higher quantity of unique microorganism than the others, which can be because of the mixed wastewater type.

**Figure 3:**
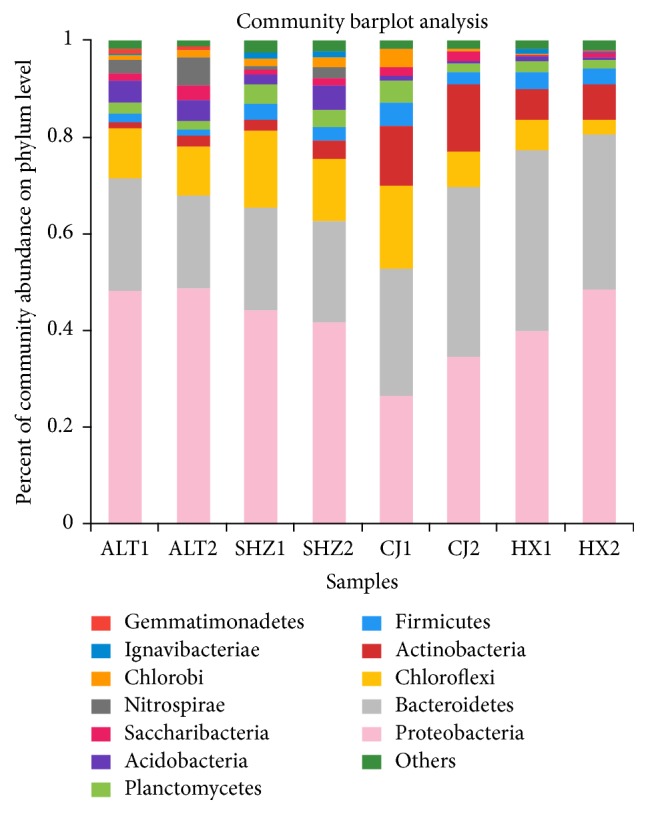
Percentages of the major phyla in all samples (the sequence percentage is above 1% in at least one sample).

A total of 36 phyla were observed in eight samples. As shown in Figure 3, Proteobacteria, which accounted for 26.7–48.9% of the classified sequences, was the most dominant phylum in all samples. Bacteroidetes, Chloroflexi, and Actinobacteria were the other important groups, comprising 19.3–37.3%, 2.9–17.1%, and 1.5–13.8% of the total sequences in each sample, respectively. These four groups accounted for 79.6–91.2% with the average of 85.3% of the total effective sequences of the eight samples. Proteobacteria was the most leading community, which is consistent with the results of the bacterial communities in soil [11, 22, 23] and activated sludge [18, 24]. Bacteroidetes, Acidobacteria, and Chloroflexi were also often found in activated sludge [25, 26]. In addition, several phyla accounted for more than 1% at least in one sample, for example, Firmicutes (1.4–4.8%), Planctomycetes (1.7–4.5%), Chlorobi (0.2–3.8%), Acidobacteria (0.5–5.1%), Saccharibacteria (0.4–3.2%), and Ignavibacteriae (0.03–1.3%).

Among Proteobacteria, Betaproteobacteria was the most abundant class (34.4–65.8%). Gammaproteobacteria was the second dominant class, accounting for 16.2 to 39.8%. Alphaproteobacteria and Deltaproteobacteria were the other important classes, comprising 11.0–38.6% and 2.2–9.1%, respectively. Epsilonproteobacteria had at the lowest abundance in the range from 0.02 to 4.3%. This result was consistent with other studies concluding that Betaproteobacteria was the largest class [18]. Within Betaproteobacteria, eleven orders were identified. Burkholderiales was the predominant main order within Betaproteobacteria between 44.1 and 82.5% of all samples. Rhodocyclales was the subdominant main order in all samples, except for ALT, in which Hydrogenophilales was the subdominant abundant order. Besides, the other eight classes with lower abundances were detected, including Methylophilales, Neisseriales, Nitrosomonadales, and Procabacteriales.

**Figure 4:**
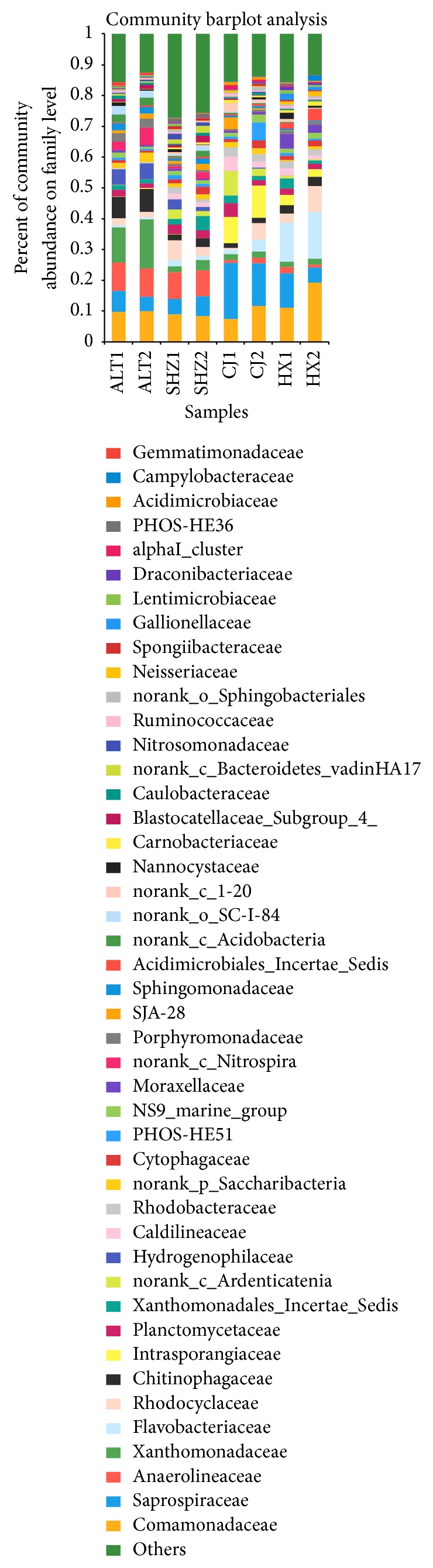
Percentages of the major families in all samples (the sequence percentage is above 1% in at least one sample).

Among the 293 families identified, 44 families including Saprospiraceae, Comamonadaceae, Anaerolineaceae, Xanthomonadaceae, Rhodocyclaceae, Flavobacteriaceae, Rhodobacteraceae, Intrasporangiaceae, Caldilineaceae,* norank_c_Ardenticatenia, norank_c_Nitrospira*, and* Xanthomonadales_Incertae_Sedis* were generally shared by all samples (>1% relative abundance at least in one sample), accounting for 71.9–87.5% of total effective sequences in each sample (Figure 4). ALT WWTP had higher abundances of Xanthomonadaceae (11.4–15.9%), Chitinophagaceae (6.9–7.5%), Hydrogenophilaceae (5.1–5.2%), and norank_c_*Nitrospira* (2.7–5.6%) compared to other samples in ranges of 1.7–3.4%, 1.5–3.0%, 0.01–3.3%, and 0.0–2.1%, respectively. Flavobacteriaceae accounted for 12.7–15.1% in HX and Intrasporangiaceae and* norank_c_Ardenticatenia* accounted for 8.6–10.5% and 2.2–8.0% in CJ, whereas their abundances were higher than other samples, in the range of 0.6–4.0% and 0.05–3.3%, 0.01–3.1%, respectively. The relative abundances of family Saprospiraceae (4.9–18.2%) were higher in bulking samples (CJ and HX WWTPs) than those in normal samples. The relative abundances of family Anaerolineaceae (8.4–9.3%) were higher in normal samples (ALT and SHZ WWTPs) than those in bulking sludge samples.

Comamonadaceae were in charge of aromatic degrading and denitrifying processes and were the chief families in numerous wastewater treatment plants [27]. The family Rhodocyclaceae contains primarily denitrifying rod-shaped or aerobic bacteria [28], which display highly multipurpose metabolic capabilities. Members of the Anaerolineae class are largely distributed in different types of natural and artificial anaerobic ecosystems, which are obligate anaerobes [29]. Filamentous Saprospiraceae and Flavobacteriaceae can cause sludge bulking [30].

**Figure 5:**
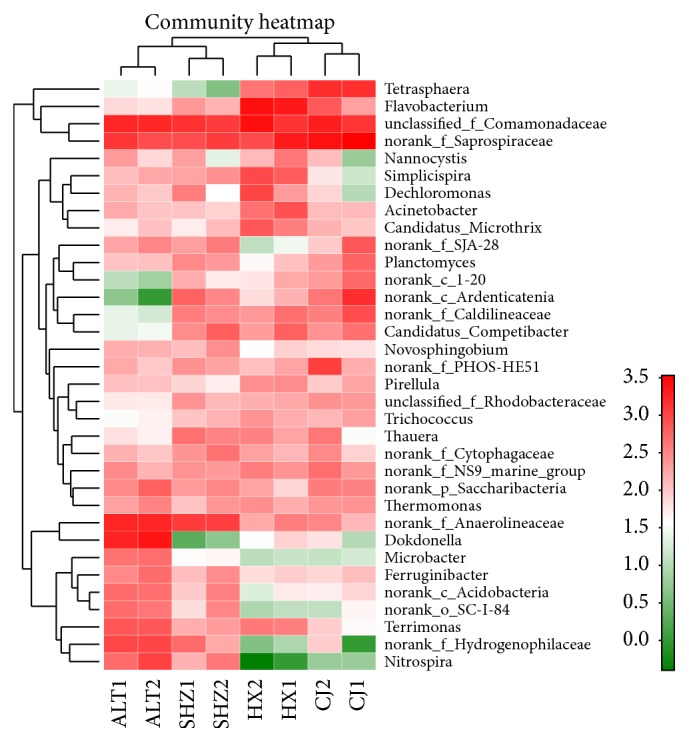
Heatmap of the first ten dominant genera in each sample.

579 genera were shared in all samples, in total. Fifty-five genera, which accounted for 60.6–82.7% of the classified sequences and included the* unclassified_f_Comamonadaceae*,* norank_f_Saprospiraceae*,* Flavobacterium*,* norank_f_Hydrogenophilaceae*,* Dokdonella*,* Terrimonas*,* norank_f_Anaerolineaceae*,* Tetrasphaera*,* Simplicispira*,* norank_c_Ardenticatenia*, and* Nitrospira* genera were generally shared by all samples. Most of them were discovered to be chief genera and were shared by activated samples from WWTPs [25]. From all samples, the first ten dominant genera in each sample were chosen (a total of 34 genera) and the comparison of their abundance was analyzed using the heatmap (Figure 5). The relative abundances of genera* Dokdonella *(11.0%),* norank_f_Hydrogenophilaceae* (5.1%),* Terrimonas *(3.8%), and* Nitrospira *(4.1%) were much higher in ALT WWTP than those in others.* norank_f_Anaerolineaceae* (averaging at 7.08%) was higher in SHZ and ALT WWTPs than those in other sludge samples (averaging at 1.43%). The samples in CJ WWTP had high levels of genus* norank_f_Saprospiraceae* (averaging at 15.5%), whereas the other samples contained relatively less (averaging at 6.3%). HX WWTP had higher abundances of* Simplicispira* (4.1%) and* Flavobacterium *(12.7%) compared to other samples with ranges of 0.1–1.3% and 0.3–3.7%, respectively. CJ and HX WWTP had higher abundances of* Tetrasphaera*, in the range of 7.7–7.9% and 3.2–2.2%, respectively, while the proportion of other samples was in ranges of 0.02–0.2%. Genus* Tetrasphaera* from family Intrasporangiaceae has a certain contribution to sludge bulking [31, 32].

Filamentous Saprospiraceae and* Tetrasphaera *multiplied had a positive impact on the sludge bulking in CJ WWTP, while filamentous* Flavobacterium *and Saprospiraceae multiplied had a positive impact on the sludge bulking in HX WWTP.

### 3.3. Relationships between Environmental Factors and Community Structure

**Figure 6:**
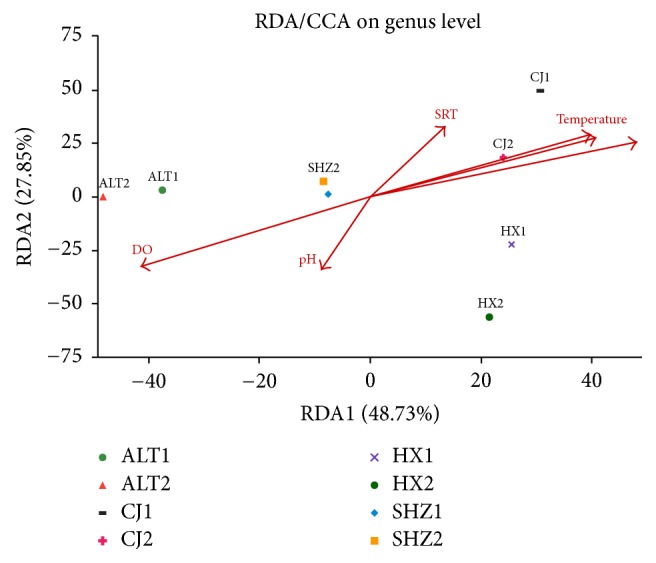
RDA analysis to investigate the relationship between microbial communities and environmental variables.

The possible relationship between microbial communities and environmental variables was analyzed using a constrained ordination of redundancy analysis (RDA). Six variables including influent BOD_5_, influent ammonia, pH, water temperature, dissolved oxygen (DO), and solid retention time (SRT) were selected, and the ordination biplot was shown in Figure 6. As evident in Figure 6, the canonical axes of first and second showed 48.73% and 27.85% of data variance, respectively. RDA indicated that influent BOD_5_, DO, water temperature, and influent ammonia had a distinct effect on bacterial community structures, though pH exhibited the least effect.

Influent BOD_5_ is the most crucial environmental factor influencing the community compositions. Previous studies have found that the types of feeding substrate have an impact on bacterial diversity [33]. Similarly, the biodegradability of influent wastewater was affected by the compositions of bacteria in the wastewater treatment [21]. The organic components are normally represented by the proxies of COD and/or biochemical oxygen demand (BOD) in wastewater. The significance of influent BOD in the process of the bacterial community formation was found in activated sludge systems, which is consistent with the result of this study [34, 35]. In this study, different WWTPs had different wastewater quality, resulting in difference in the wastewater constituents. Therefore, influent BOD_5_ can possibly interpret the difference of bacterial communities.

DO had a significant influence on the bacterial communities. In the context of its influence on microbial activity and the high operating costs of aeration, DO is a key operational parameter in wastewater treatment systems. However, the explicit selection by DO concentration is not completely understood for diverse bacterial lineages. The results showed that DO had an important influence for shaping the compositions of the microbial community of wastewater treatment processes. Several studies have found that DO concentration was a significant structuring factor for bacterial community compositions running at high and low DO concentrations in bioreactors of two laboratory scales [36].

The water temperature was correlated with variance of the bacterial community, which is in agreement with other studies [24, 37]. Some studies have shown that bacterial community structures were influenced significantly by the influent ammonia, which disagrees with the result of this study [38].

In this study, pH was found to have the least impact on bacterial community structures, which is in disagreement with the other studies [18]. Several studies have illustrated that the whole diversity and structures of microbial communities were affected by the pH in a series of aquatic and terrestrial environments [39, 40]. In the present study, it might not be generated on the whole microbial communities, because pH did not change significantly among these WWTPs (Table 1). Thus, further investigation is needed to understand this aspect.

## 4. Conclusions

In conclusion, 36 phyla, 293 families, and 579 genera were found in activated sludge samples from WWTPs of Xinjiang in winter. The number of shared OTUs accounted for 26.6% of the total observed OTUs; it can be concluded that some microorganisms always existed in the activated sludge collected from different WWTPs. The WWTP with low temperature and single wastewater type was found to have the lowest microbial diversity. The WWTP with mixed wastewater type was found to have the highest microbial diversity. Proteobacteria, Bacteroidetes, Chloroflexi, and Acidobacteria, accounting for 79.6–91.2% of the classified sequences, were the most abundant phyla in all samples. Fifty-five genera, which accounted for 60.6–82.7% of the classified sequences and included* unclassified_f_Comamonadaceae*,* norank_f_Saprospiraceae*,* Flavobacterium*,* norank_f_Hydrogenophilaceae*,* Dokdonella*,* Terrimonas*,* norank_f_Anaerolineaceae*,* Tetrasphaera*,* Simplicispira*,* norank_c_Ardenticatenia*, and* Nitrospira* genera, were generally shared by all samples. The relative abundances of Saprospiraceae,* Flavobacterium*, and* Tetrasphaera* in bulking sludge samples were much higher than that in normal samples. Filamentous Saprospiraceae,* Flavobacterium*, and* Tetrasphaera* multiplied were the main cause for the sludge bulking. Redundancy analysis (RDA) indicated that influent BOD_5_, DO, water temperature, and influent ammonia had a significant impact on bacterial community compositions, whereas pH exhibited the least influence.

## Figures and Tables

**Table 1:** Characteristics of samples and wastewater treatment plants.

Sample	Sampling date	Flow rate (10^3^ m^3^/d)	DO (mg/L)	pH	SRT (days)	Tem (°C)	SV/%	MLSS (mg/L)	SVI/(mL/g)	BOD_5_ (mg/L)		NH_4_^+^-N (mg/L)	
										Influent	Effluent	Influent	Effluent
ALT1	2016.12.1	3	3.8	7.0	27	9.8	35	5980	59	119	8	33.1	1.6
ALT2	2017.1.7	3	3.9	7.1	27	8.5	33	4861	69	97	9	29.7	2.0
SHZ1	2016.3.2	10	4.0	7.50	23	12.8	17	4186	41	147	39	28	3.0
SHZ2	2017.1.7	10	3.7	7.4	23	12.9	19	4297	44	164	30	27	3.2
CJ1	2016.1.25	10	1.5	6.75	30	13.6	88	3355	262	539	27	58	21.9
CJ2	2016.3.2	10	1.4	7.01	30	13.5	91	4512	202	533	25	47	20.6
HX1	2016.1.25	10	3.6	7.20	25	10.8	88	4354	199	276	11	50	5.8
HX2	2016.3.2	10	3.5	7.30	25	10.9	62	3543	175	276	26	31	5.2

**Table 2:** Richness and diversity indices of microbial communities for sludge samples.

Sample	Reads	OTUs	Shannon	Ace	Chao	Coverage
ALT1	32390	714	4.88	811	802	0.992
ALT2	38394	683	4.52	863	908	0.989
SHZ1	53363	1152	5.93	1292	1281	0.988
SHZ2	30087	1022	5.78	1200	1192	0.988
CJ1	46680	852	5.09	1054	1047	0.988
CJ2	55170	855	5.16	1031	1024	0.989
HX1	48497	829	5.13	1003	994	0.989
HX2	52873	755	4.76	909	905	0.990

## References

[1] Wagner M., Loy A. (2002). Bacterial community composition and function in sewage treatment systems. *Current Opinion in Biotechnology*.

[2] Miura Y., Hiraiwa M. N., Ito T., Itonaga T., Watanabe Y., Okabe S. (2007). Bacterial community structures in MBRs treating municipal wastewater: Relationship between community stability and reactor performance. *Water Research*.

[3] Jenkins D. (1993). *Manual on the Causes and Control of Activated Sludge Bulking and Foaming*.

[4] Martins A. M. P., Pagilla K., Heijnen J. J., Van Loosdrecht M. C. M. (2004). Filamentous bulking sludge—a critical review. *Water Research*.

[5] Kragelund C., Levantesi C., Borger A. (2008). Identity, abundance and ecophysiology of filamentous bacteria belonging to the Bacteroidetes present in activated sludge plants. *Microbiology*.

[6] Liu Z. C., Li F., Li Q. (2015). Effect of ozone on filamentous bulking under low temperature. *China Water & Wastewater*.

[7] Glenn T. C. (2011). Field guide to next-generation DNA sequencers. *Molecular Ecology Resources*.

[8] Albertsen M., Hansen L. B. S., Saunders A. M., Nielsen P. H., Nielsen K. L. (2012). A metagenome of a full-scale microbial community carrying out enhanced biological phosphorus removal. *Isme Journal Multidisciplinary Journal of Microbial Ecology*.

[9] Ju F., Guo F., Ye L., Xia Y., Zhang T. (2014). Metagenomic analysis on seasonal microbial variations of activated sludge from a full-scale wastewater treatment plant over 4 years. *Environmental Microbiology Reports*.

[10] Qian P.-Y., Wang Y., Lee O. O. (2011). Vertical stratification of microbial communities in the Red Sea revealed by 16S rDNA pyrosequencing. *The ISME Journal*.

[11] Roesch L. F. W., Fulthorpe R. R., Riva A. (2007). Pyrosequencing enumerates and contrasts soil microbial diversity. *The ISME Journal*.

[12] Claesson M. J., O'Toole P. W. (2014). Evaluating the latest high-throughput molecular techniques for the exploration of microbial gut communities. *Gut Microbes*.

[13] Xia S., Duan L., Song Y. (2010). Bacterial community structure in geographically distributed biological wastewater treatment reactors. *Environmental Science & Technology*.

[14] Hu Q.-Y., Li M., Wang C., Ji M. (2015). Influence of powdered activated carbon addition on water quality, sludge properties, and microbial characteristics in the biological treatment of commingled industrial wastewater. *Journal of Hazardous Materials*.

[15] Kim Y. M., Cho H. U., Lee D. S., Park D., Park J. M. (2011). Influence of operational parameters on nitrogen removal efficiency and microbial communities in a full-scale activated sludge process. *Water Research*.

[16] Shu D., He Y., Yue H., Wang Q. (2015). Microbial structures and community functions of anaerobic sludge in six full-scale wastewater treatment plants as revealed by 454 high-throughput pyrosequencing. *Bioresource Technology*.

[17] Wagner M., Loy A., Nogueira R., Purkhold U., Lee N., Daims H. (2002). Microbial community composition and function in wastewater treatment plants. *Antonie van Leeuwenhoek*.

[18] Gao P., Xu W., Sontag P. (2016). Correlating microbial community compositions with environmental factors in activated sludge from four full-scale municipal wastewater treatment plants in Shanghai, China. *Applied Microbiology and Biotechnology*.

[19] Peng Y. Z., Gao J. H. Mechanism, cause and control of activated sludge expansion.

[20] Lettinga G., Rebac S., Zeeman G. (2001). Challenge of psychrophilic anaerobic wastewater treatment. *Trends in Biotechnology*.

[21] Ibarbalz F. M., Figuerola E. L. M., Erijman L. (2013). Industrial activated sludge exhibit unique bacterial community composition at high taxonomic ranks. *Water Research*.

[22] Spain A. M., Krumholz L. R., Elshahed M. S. (2009). Abundance, composition, diversity and novelty of soil *Proteobacteria*. *The ISME Journal*.

[23] Sun M., Xiao T., Ning Z., Xiao E., Sun W. (2015). Microbial community analysis in rice paddy soils irrigated by acid mine drainage contaminated water. *Applied Microbiology and Biotechnology*.

[24] Wang X., Hu M., Xia Y., Wen X., Ding K. (2012). Pyrosequencing analysis of bacterial diversity in 14 wastewater treatment systems in China. *Applied and Environmental Microbiology*.

[25] Zhao D., Huang R., Zeng J. (2014). Pyrosequencing analysis of bacterial community and assembly in activated sludge samples from different geographic regions in China. *Applied Microbiology and Biotechnology*.

[26] Zhang T., Shao M.-F., Ye L. (2012). 454 Pyrosequencing reveals bacterial diversity of activated sludge from 14 sewage treatment plants. *The ISME Journal*.

[27] Loy A., Schulz C., Lücker S. (2005). 16S rRNA gene-based oligonucleotide microarray for environmental monitoring of the betaproteobacterial order ‘Rhodocyclales’. *Applied and Environmental Microbiology*.

[28] Kuever J. F. A. R., Widdel F. (2005). *Bergey's Manual of Systematic Bacteriology, the Proteobacteria. Part C, the Alpha-, Beta-, Delta-, and Epsilonproteobacteria*.

[29] Rosenkranz F., Cabrol L., Carballa M. (2013). Relationship between phenol degradation efficiency and microbial community structure in an anaerobic SBR. *Water Research*.

[30] Duan Z. H., Pan P. L. M., Chen C. X. O., Wang X. D., Zhao L. J., Tian L. Q. (2016). Changes of microbial community structure in activated sludge bulking at low temperature. *Environmental Science*.

[31] McKenzie C. M., Seviour E. M., Schumann P. (2006). Isolates of “Candidatus Nostocoida limicola” Blackall et al. 2000 should be described as three novel species of the genus Tetrasphaera, as Tetrasphaera jenkinsii sp. nov., Tetrasphaera vanveenii sp. nov. and Tetrasphaera veronensis sp. nov. *International Journal of Systematic and Evolutionary Microbiology*.

[32] Wang P., Yu Z., Qi R., Zhang H. (2016). Detailed comparison of bacterial communities during seasonal sludge bulking in a municipal wastewater treatment plant. *Water Research*.

[33] Pholchan M. K., Baptista J. D. C., Davenport R. J., Curtis T. P. (2010). Systematic study of the effect of operating variables on reactor performance and microbial diversity in laboratory-scale activated sludge reactors. *Water Research*.

[34] Kim B.-C., Kim S., Shin T., Kim H., Sang B.-I. (2013). Comparison of the bacterial communities in anaerobic, anoxic, and oxic chambers of a pilot A_2_O process using pyrosequencing analysis. *Current Microbiology*.

[35] Van Der Gast C. J., Ager D., Lilley A. K. (2008). Temporal scaling of bacterial taxa is influenced by both stochastic and deterministic ecological factors. *Environmental Microbiology*.

[36] Park H.-D., Noguera D. R. (2004). Evaluating the effect of dissolved oxygen on ammonia-oxidizing bacterial communities in activated sludge. *Water Research*.

[37] Wells G. F., Park H.-D., Yeung C.-H., Eggleston B., Francis C. A., Criddle C. S. (2009). Ammonia-oxidizing communities in a highly aerated full-scale activated sludge bioreactor: Betaproteobacterial dynamics and low relative abundance of Crenarchaea. *Environmental Microbiology*.

[38] Ma Q., Qu Y., Shen W. (2015). Bacterial community compositions of coking wastewater treatment plants in steel industry revealed by Illumina high-throughput sequencing. *Bioresource Technology*.

[39] Hörnström E. (2002). Phytoplankton in 63 limed lakes in comparison with the distribution in 500 untreated lakes with varying pH. *Hydrobiologia*.

[40] Fierer N., Jackson R. B. (2006). The diversity and biogeography of soil bacterial communities. *Proceedings of the National Acadamy of Sciences of the United States of America*.

